# Supplementary material for: Methodological recommendations for assessing scleractinian and octocoral recruitment to settlement tiles
Source: PeerJ. 2021 Dec 17;9:e12549. doi: 10.7717/peerj.12549 (PMC8686733; doi:10.7717/peerj.12549)
Supplement: Supplemental Information 1 — Recruit density reflects total scleractinian recruitment unless otherwise specified, and densities and percentages are reported as a range by site-deployment wherever possible. These examples illustrate difference methodological choices made during scleractinian recruitment studies and the discrepancies in reporting convention that may render interstudy comparisons problematic. [file peerj-09-12549-s001.docx]

**Table S1. Selected recruitment studies from 2007-2020 (ordered chronologically).** Recruit density reflects total scleractinian recruitment unless otherwise specified, and densities and percentages are reported as a range by site-deployment wherever possible. These examples illustrate difference methodological choices made during scleractinian recruitment studies and the discrepancies in reporting convention that may render interstudy comparisons problematic.

| **Citation** | **Location** | **Years of deployment** | **Number of sites** | **Tiles per site** | **Depth range (m)** | **Surfaces surveyed** | **Length of deployments** | **Number of deployments** | **Tiles bleached (yes/no)** | **Recruit density (recruits/m^2^)** | **Overall percent of recruits on top surfaces** |
| --- | --- | --- | --- | --- | --- | --- | --- | --- | --- | --- | --- |
| Adjeroud, Penin, & Carroll, 2007 | Moorea, French Polynesia | 2001-2003 | 9 | 20 | 6-18 | all | 3 months | 12 | not specified | 4.56 - 24.67 | 14.5% |
| Burt et al. 2009 | Abu Dhabi, UAE | 2007-2008 | 4 | 125 | 8 | bottom | 12 months | 1 | yes | 10 - 490 | NA |
| Arnold, Steneck, & Mumby, 2010 | Bonaire | 2004-2006 | 6 | 40 | 10 | bottom | 27 months (analyzed 6 times during deployment) | 1 (multiple scans of same tiles) | no | 50 - 175 | NA |
| Arnold and Steneck 2011 | Carrie Bow Cay, Belize | 2005-2008 | 4 | 25 | 10 | bottom | 3.17 years (analyzed 5 times during deployment) | 1 (multiple scans of same tiles) | no | 0.3 - 226 | NA |
| Green and Edmunds 2011 | St John | 2006-2007 | 10 | 10-15 | 5-6 | all | 6 months | 4 | yes | 59.6 | 0% |
| Chong-Seng, Graham, & Pratchett 2014 | Seychelles | 2012 | 9 | 10 | 4 | bottom | 3 months | 1 | yes | 166-2166 | NA |
| Doropoulos et al. 2014 | Palau | 2013 | 6 | 15 | 7 | all | 1.5 months (during spawning) | 1 | no | 0 - 7560 | top density was 13% of bottom density |
| Edmunds, Nozawa, & Villanueva, 2014 | St John | 2010-2012 | 5 | 15 | 5 | all | 12 months | 2 | yes | 14 - 117 | 6% |
| Humanes and Bastidas 2015 | Los Roques, Venezuela | 2007-2008 | 4 | 15 | 3-5 | all; bottom surface excluded from rate calculations | 5-6 months | 4 | no | 30 - 236 | 0 |
| Edmunds 2017 | Moorea, French Polynesia | 2005-2015 | 23 | 15 | 2-17 | all | 6 months | 20 | yes | 4.7 - 219.6 | <20% |
| Davidson et al. 2019 | Great Barrier Reef | 2006-2012 | 24 | 18 | 5 | all | 2-3 months (during spawning) | 10 | yes | 1065 (only acroporids counted) | not specified (51% on side) |
| Jouval et al. 2019 | Reunion and Rodrigues | 2017-2017 | 8 | 20 | 12 | all | 6 months | 3 | yes | 0 - 150 | majority on sides |
| Burt and Bauman 2020 | Abu Dhabi, UAE | 2010-2012 | 10 | 25 | 4-6 | bottom | 3 months | 8 | yes | 40 - 95 | NA |
| **This study** | Florida, United States | 2015-2018 | 30 | 32 | 1.5 - 18 | all | 12 months | 3 | yes | scleractinian:  1.9 – 1587.8  octocoral:  0-122.6 | scleractinian:  83.5%  octocoral 66.7% |
