# Supplementary material for: Methodological recommendations for assessing scleractinian and octocoral recruitment to settlement tiles
Source: PeerJ. 2021 Dec 17;9:e12549. doi: 10.7717/peerj.12549 (PMC8686733; doi:10.7717/peerj.12549)
Supplement: Supplemental Information 2 — Dates (dd/mm/yyyy) for each year (Yr 1–3) of tile deployment and retrieval are shown. [file peerj-09-12549-s002.docx]

**Table S2.** **Characteristics of study sites (listed north to south).** Site numbers correspond with those displayed in Fig 1. Dates (dd/mm/yyyy) for each year (Yr 1-3) of tile deployment and retrieval are shown.

| Site No. | Site Name | Region | Reef Type | Depth (m) | Latitude (DD) | Longitude (DD) | Yr 1 Deployment | Yr 1 Retrieval & Yr 2 Deployment | Yr 2 Retrieval & Yr 3 Deployment | Yr 3 Retrieval |
| --- | --- | --- | --- | --- | --- | --- | --- | --- | --- | --- |
| 1 | BC2 | Broward County | Middle reef | 12.0 | 26.1599 | -80.0825 | 03/04/2015 | 01/04/2016 | 09/02/2017 | 26/02/2018 |
| 2 | BC3 | Broward County | Outer reef | 16.5 | 26.1586 | -80.0773 | 30/03/2015 | 05/05/2016 | 21/03/2017 | 28/02/2018 |
| 3 | BC4 | Broward County | Inner reef | 7.5 | 26.1494 | -80.0894 | 20/03/2015 | 29/03/2016 | 08/02/2017 | 28/02/2018 |
| 4 | BC1 | Broward County | Nearshore | 7.5 | 26.1479 | -80.0959 | 19/03/2015 | 17/03/2016 | 07/02/2017 | 26/02/2018 |
| 5 | DC7 | Dade County | Middle reef | 18.0 | 25.9585 | -80.0938 | 02/04/2015 | 23/05/2016 | 27/03/2017 | 13/03/2018 |
| 6 | DC6 | Dade County | Nearshore | 6.0 | 25.9516 | -80.1091 | 01/04/2015 | 11/05/2016 | 14/03/2017 | 28/02/2018 |
| 7 | DC1 | Dade County | Inner reef | 7.5 | 25.8422 | -80.1040 | 27/04/2015 | 14/04/2016 | 13/02/2017 | 28/02/2018 |
| 8 | DC3 | Dade County | Outer reef | 16.5 | 25.8421 | -80.0881 | 24/04/2015 | 11/05/2016 | 29/03/2017 | 02/03/2018 |
| 9 | DC2 | Dade County | Middle reef | 13.5 | 25.8420 | -80.0951 | 27/04/2015 | 25/04/2016 | 14/02/2017 | 02/03/2018 |
| 10 | DC8 | Dade County | Nearshore | 4.5 | 25.6785 | -80.1186 | 17/04/2015 | 13/04/2016 | 17/02/2017 | 16/03/2018 |
| 11 | DC4 | Dade County | Outer reef | 12.3 | 25.6726 | -80.0884 | 15/04/2015 | 04/05/2016 | 28/03/2017 | 15/03/2018 |
| 12 | DC5 | Dade County | Inner reef | 7.2 | 25.6518 | -80.0946 | 23/03/2015 | 12/05/2016 | 22/03/2017 | 27/02/2018 |
| 13 | Carysfort Deep | Upper Keys | Forereef deep | 15.6 | 25.2208 | -80.2099 | 17/03/2015 | 17/03/2016 | 13/03/2017 | 11/04/2018 |
| 14 | Carysfort Shallow | Upper Keys | Forereef shallow | 2.4 | 25.2201 | -80.2105 | 17/03/2015 | 17/03/2016 | 13/03/2017 | 11/04/2018 |
| 15 | Porter Patch | Upper Keys | Patch | 4.8 | 25.1032 | -80.3243 | 18/03/2015 | 14/03/2016 | 10/03/2017 | 11/04/2018 |
| 16 | Admiral | Upper Keys | Patch | 1.5 | 25.0447 | -80.3948 | 16/03/2015 | 18/03/2016 | 12/03/2017 | 10/04/2018 |
| 17 | Molasses Shallow | Upper Keys | Forereef shallow | 6.6 | 25.0088 | -80.3765 | 15/03/2015 | 15/03/2016 | 11/03/2017 | 10/04/2018 |
| 18 | Molasses Deep | Upper Keys | Forereef deep | 13.5 | 25.0072 | -80.3756 | 16/03/2015 | 17/03/2016 | 25/04/2017 | 10/04/2018 |
| 19 | Tennessee Deep | Middle Keys | Forereef deep | 13.2 | 24.7527 | -80.7578 | 19/03/2015 | 25/03/2016 | 21/03/2017 | 04/04/2018 |
| 20 | Tennessee Shallow | Middle Keys | Forereef shallow | 6.3 | 24.7450 | -80.7812 | 19/03/2015 | 25/03/2016 | 22/03/2017 | 04/04/2018 |
| 21 | West Turtle Shoal | Middle Keys | Patch | 6.6 | 24.6993 | -80.9669 | 20/03/2015 | 26/03/2016 | 26/04/2017 | 02/04/2018 |
| 22 | Dustan Rocks | Middle Keys | Patch | 4.5 | 24.6895 | -81.0302 | 20/03/2015 | 19/03/2016 | 01/03/2017 | 02/04/2018 |
| 23 | Sombrero Shallow | Middle Keys | Forereef shallow | 5.1 | 24.6253 | -81.1116 | 21/03/2015 | 28/03/2016 | 02/03/2017 | 31/03/2018 |
| 24 | Sombrero Deep | Middle Keys | Forereef deep | 15.0 | 24.6223 | -81.1120 | 21/03/2015 | 28/03/2016 | 26/04/2017 | 09/04/2018 |
| 25 | West Washerwoman | Lower Keys | Patch | 7.5 | 24.5475 | -81.5866 | 27/02/2015 | 07/04/2016 | 02/03/2017 | 07/04/2018 |
| 26 | Red Dun Reef | Lower Keys | Patch | 7.8 | 24.5036 | -81.7677 | 24/02/2015 | 29/03/2016 | 21/03/2017 | 07/04/2018 |
| 27 | Western Sambo Shallow | Lower Keys | Forereef shallow | 4.2 | 24.4796 | -81.7176 | 25/02/2015 | 29/03/2016 | 02/03/2017 | 05/04/2018 |
| 28 | Western Sambo Deep | Lower Keys | Forereef deep | 12.0 | 24.4780 | -81.7171 | 22/03/2015 | 29/03/2016 | 02/03/2017 | 05/04/2018 |
| 29 | Sand Key Shallow | Lower Keys | Forereef shallow | 6.3 | 24.4520 | -81.8775 | 25/02/2015 | 28/03/2016 | 22/03/2017 | 06/04/2018 |
| 30 | Sand Key Deep | Lower Keys | Forereef deep | 10.5 | 24.4517 | -81.8798 | 23/02/2015 | 25/03/2016 | 22/03/2017 | 06/04/2018 |
